# Unravelling the mechanisms of aducanumab on efficacy of Aß clearance by iPSC‐derived human microglia in 2D and 3D cell cultures

**DOI:** 10.1002/alz.091108

**Published:** 2025-01-03

**Authors:** Ada J. Lin, Stefan Wendt, Declan Brennan, Haakon B. Nygaard

**Affiliations:** ^1^ University of British Columbia, Vancouver, BC Canada

## Abstract

**Background:**

Antibodies targeting amyloid beta (Aß) have recently been developed as promising treatments for Alzheimer’s Disease (AD). Aducanumab was the first to receive limited FDA approval in 2021 offering much needed new treatment options for AD patients. Aducanumab clears aggregated Aß in the brain, presumably through microglial phagocytosis, although the exact cellular and molecular mechanisms of its effect remain incompletely understood. We employed iPSC‐derived microglia in 2D and 3D cultures to study the mechanisms by which aducanumab facilitates Aß clearance in the brain.

**Method:**

iPSC‐derived microglia were studied in both 2D and 3D environments. Microglia plated in 2D were treated with pHrodo conjugated oligomeric Aß42 and aducanumab and imaged live for 24 hours to monitor short term changes. Cells and supernatant were collected for ELISA afterwards. For 3D cell culture, neurospheres were formed from iPSC‐derived neuronal progenitor cells, which grew to consist of mature neurons and astrocytes. Microglia were added to the neurospheres and infiltrated into the 3D tissue, followed by longer Aß and aducanumab treatment. Both the neurospheres and the surrounding 2D microglia were imaged afterwards, and the supernatant was collected for Aß ELISA.

**Result:**

Live imaging 2D microglia over 24 hours indicated that microglia treated with aducanumab have significantly higher levels of phagocytosed Aß and at a faster rate. Intriguingly, immunostaining for fibrillar Aß revealed that aducanumab decreased the amount of highly aggregated intracellular Aß. In the neurospheres, microglia are the primary cell type phagocytosing Aß. Aducanumab did not cause a significant reduction of Aß in the 3D tissue but prevented the formation of larger Aß aggregates, which may in turn facilitate microglia mediated Aß clearance.

**Conclusion:**

Aducanumab acutely increases the ability of iPSC‐derived microglia to phagocytose Aß and may also prevent Aß aggregation. 3D neurospheres can be used to study more functional impacts of aducanumab on neuronal function. Further analysis of phagocytosed Aß using biochemical techniques after anti‐Aß antibody treatment will help to determine the precise molecular mechanisms of its effects.

